# Supplementary material for: Arboviral Disease Outbreaks in the Pacific Islands Countries and Areas, 2014 to 2020: A Systematic Literature and Document Review
Source: Pathogens. 2022 Jan 7;11(1):74. doi: 10.3390/pathogens11010074 (PMC8779081; doi:10.3390/pathogens11010074)
Supplement: Supplementary file 1 [file pathogens-11-00074-s001.zip › pathogens-1513115-supplementary/Supplementary files/Suppl material_File S3.pdf]

Supplementary material

File S3. Literature search strategy (OVID - Medline)

Pacific islands/ OR ("pacific region" OR "pacific Island*" OR "pacific-island*" OR "american samoa" OR "Cook island*" OR "federated states of micronesia" OR micronesia OR Yap OR Chuuk OR Pohnpei OR Kosrae OR Fiji OR "Fiji Island*" OR "French Polynesia" OR Polynesia OR Guam OR Kiribati OR "Marshall Island*" OR Nauru OR "New Caledonia" OR Niue OR "Northern Mariana Island*" OR "Commonwealth of the Northern Marina Island*" OR Palau OR "Papua New Guinea" OR "Pitcairn Island*" OR Samoa OR "Solomon Island*" OR Tokelau OR Tonga OR Vanuatu OR "Wallis and Futuna" OR Wallis OR Futuna OR "États Fédérés de Micronésie" OR "Îles Cook" OR "Îles Fidji" OR "Îles Mariannes du Nord" OR "Îles Marshall" OR "Îles Pitcairn" OR "Îles Salomon" OR Nioué OR "Nouvelle-Calédonie" OR "Nouvelle Calédonie" OR "Papouasie Nouvelle Guinée" OR "Papouasie-Nouvelle-Guinée" OR "Polynésie française" OR "Samoa américaines" OR "Wallis et Futuna").

AND

Arboviruses/ OR Arboviral infections/ OR Flavivirus/ OR (Arbovir* OR "arthopod-borne virus*" OR "arthopod borne virus*" OR "Mosquito-borne virus*" OR "mosquito borne virus*" OR "mosquito borne disease" OR "mosquito-borne disease" OR "Vector-borne virus*" OR "Vector borne virus*" OR Flavivirus OR Flaviviridae OR alphavirus OR togaviridae OR dengue OR "Dengue Infection*" OR "Dengue Virus*" OR Zika OR "Zika virus*" OR Chikungunya OR "japanese encephalitis virus" OR "encéphalite japonaise" OR "ross river virus" OR "West nile virus" OR "Yellow fever virus" OR "Fièvre jaune" OR "barmah forest virus" OR "Murray valley encephalitis" OR "Kinjin" OR "Sinbis virus").

AND

Disease Outbreaks/ OR Disease outbreaks/ OR Epidemics/ OR Endemic Diseases/ OR Disease Transmission, Infectious/ OR (Outbreak OR epidemic OR endemic OR cluster OR épidémie OR endémique)
