# Supplementary material for: Arboviral Disease Outbreaks in the Pacific Islands Countries and Areas, 2014 to 2020: A Systematic Literature and Document Review
Source: Pathogens. 2022 Jan 7;11(1):74. doi: 10.3390/pathogens11010074 (PMC8779081; doi:10.3390/pathogens11010074)
Supplement: Supplementary file 1 [file pathogens-11-00074-s001.zip › pathogens-1513115-supplementary/Supplementary files/Suppl material_Table S1.pdf]

Supplementary material

Table S1. Summary of arboviral disease outbreaks (confirmed/ suspected/ unspecified) affecting Pacific island countries and areas, October 1, 2014 to June 30, 2020.

First reported (Mth/ Yr)	PICs	Latest information	Implicated virus	Summary notes and reported case numbers (confirmed/suspected/unspecified)
Pre-Oct 2014	New Caledonia	Nov 2014	DENV unspecified	PacNet: Not mentioned on PacNet during our date range<br>Literature: Not described in the literature<br>ProMed: 334 cases reported<br>The majority of this outbreak occurred prior to the study date range<br>Imported cases: No imported cases were reported in another country from this outbreak
Pre-Oct 2014	Tonga	Mar 2015	DENV3	PacNet: 174 cases reported to PacNet - Stated DENV3 was identified<br>Literature: Craig 2018 reported that there was an outbreak of DEN3 during this time, however, did not mention specific case numbers<br>ProMed: 400 cases mentioned on ProMed<br>Imported cases: 24 cases detected in New Zealand as imported cases from Tonga
Pre-Oct 2014	French Polynesia	May 2016	DENV1	PacNet: 1114 cases reported to PacNet - reported that it was DENV1<br>Literature: Aubry 2019mentioned described this outbreak however did not report specific numbers<br>Craig 2018 reported that there was an outbreak of DEN1 during this time, however, did not mention specific case numbers<br>ProMed: 2188 cases reported on ProMed<br>Imported cases: 14 cases detected as imported cases into New Zealand, 1 imported case into the Cook Islands
Nov 2014	Cook Islands	Feb 2015	DENV1	PacNet: 2 cases confirmed as DENV1<br>Literature: Not described in the literature<br>ProMed: Not reported on ProMed<br>Imported cases: No imported cases were reported in another country from this outbreak
Nov 2014	Cook Islands	Feb 2015	DENV3	PacNet: 1 case confirmed as DENV3<br>Literature: Not described in the literature<br>ProMed: Not reported on ProMed<br>Imported cases: No imported cases were reported in another country from this outbreak
Nov 2014	Cook Islands	Feb 2015	DENV unspecified	PacNet: 2 cases identified with an unspecified serotype<br>Literature: Not described in the literature

First reported (Mth/ Yr)	PICs	Latest information	Implicated virus	Summary notes and reported case numbers (confirmed/suspected/unspecified)
				ProMed: Not reported on ProMed<br>Imported cases: No imported cases were reported in another country from this outbreak
Jan 2015	Fiji	Feb 2015	DENV2	PacNet: 157 cases reported<br>Literature: Craig 2018 reported that there was an outbreak of DEN2 during this time; however, it did not mention specific case numbers<br>ProMed: 3 articles mentioned outbreaks in Fiji on ProMed, all with varying case numbers ranging from 382 to 500<br>Imported cases: 6 cases were imported into New Zealand as imported cases from Fiji
Feb 2015	New Caledonia	May 2015	DENV unspecified	PacNet: 3 autochthonous cases reported on Pacnet<br>Literature: Not described in the literature<br>ProMed: 2 ProMed articles mention this outbreak. The first described 1 local case; however, the other reported 200 cases<br>Imported cases: No imported cases were reported in another country from this outbreak
Feb 2015	Kiribati	Feb 2015	DENV unspecified	PacNet: There was an entry on PacNet describing this outbreak; however, it was only reported as "several" cases<br>Literature: Not described in the literature<br>ProMed: Not reported on ProMed<br>Imported cases: No imported cases were reported in another country from this outbreak
March 2015	Solomon Islands	Mar 2015	DENV3	PacNet: 9 laboratory-confirmed cases<br>Literature: Not described in the literature<br>ProMed: Not reported on ProMed<br>Imported cases: No cases were imported into another country
May 2015	America Samoa	Nov 2015	DENV3	PacNet: 477 cases<br>Literature: Cotter 2019 described this outbreak with total numbers reaching 900 cases<br>Craig 2018 reported that there was an outbreak of DENV3 during this time, however, it did not mention specific case numbers<br>ProMed: Not reported on ProMed<br>Imported cases: No cases were imported into another country
June 2015	Samoa	Sept 2016	DENV3	PacNet: 1507 cases<br>Literature: Craig 2018 reported that there was an outbreak of DENV3 during this time, however, did not mention

First reported (Mth/ Yr)	PICs	Latest information	Implicated virus	Summary notes and reported case numbers (confirmed/suspected/unspecified)
				specific case numbers<br>ProMed: Not reported on ProMed<br>Imported cases: 31 cases from this outbreak were imported to New Zealand, 21 to Queensland, Australia Australia
Oct 2015	New Caledonia	Oct 2015	DENV1	PacNet: 1 case<br>Literature: Not described in the literature<br>ProMed: Not reported on ProMed<br>Imported cases: No cases were imported into another country
Dec 2015	PNG	May 2016	DENV2	Pacnet: DENV2 was reported as an outbreak in Dec 2015 in Western Province of PNG<br>Literature: 2 literature sources discuss DENV2 during this time frame in PNG<br>Moore 2017: Discussed increased activity of DENV2 in 2015 and 2016<br>Craig 2018 reported that there was an outbreak of DENV2 during this time; however, it did not mention specific case numbers<br>ProMed: One article reported 250 cases total of all serotypes. One article reported 48 imported DENV2 cases into Queensland, Australia<br>Imported cases reported on PacNet: 57 cases of DENV2 imported into Queensland, Australia, Australia
Jan 2016	PNG	May 2016	DENV1	PacNet: PacNet did not report local DENV1 cases during this period; however, it did report DENV2, DENV3 and unspecified, and it did report imported cases of DENV1 from PNG to Queensland, Australia, Australia<br>Literature: 2 literature sources discuss DENV1 during this time frame in PNG<br>Moore 2017: Reported DENV1 activity in 2016<br>ProMed: One article reported 250 cases total of all serotypes. One article reported 5 imported DENV1 cases into Queensland, Australia<br>Imported cases reported on PacNet: 5 cases of DENV1 imported into Queensland, Australia, Australia
Jan 2016	PNG	May 2016	DENV3	PacNet: Circulation of DENV3 was reported on PacNet in May 2016<br>Literature: 3 literature sources discuss DENV3 during this time frame in PNG<br>Mavian 2018 states that DENV3 was present in PNG in 2016, nil specific case numbers discussed<br>Moore 2017:Reported DENV3 activity in 2016<br>ProMed: One article reported 250 cases total of all serotypes. One article reported 3 imported DENV3 cases into Queensland, Australia

First reported (Mth/ Yr)	PICs	Latest information	Implicated virus	Summary notes and reported case numbers (confirmed/suspected/unspecified)
				Imported cases reported on PacNet: 4 cases of DENV3 imported into Queensland, Australia, Australia
Jan 2016	PNG	October 2016	DENV4	PacNet: PacNet did not report local DENV4 cases during this period; however, it did report DENV2, DENV3 and unspecified, and it did report imported cases of DENV4 from PNG to Queensland, Australia, Australia<br>Literature: Moore 2017 reported samples of DENV4 recorded between July and October 2016<br>ProMed: One article reported 250 cases total of all serotypes. One article reported 1 imported DENV4 case into Queensland, Australia<br>Imported cases reported on PacNet: 4 cases of DENV4 imported into Queensland, Australia, Australia
Jan 2016	PNG	Oct 2016	DENV unspecified	PacNet: 170 DENV unspecified cases in Jan 2016<br>Literature: 2 literature sources discussed DENV unspecified at this time<br>Pulsan 2016 studied a specific cohort of 165 children admitted to hospital with DENV from Jan-July 2016 with no mention of a specific serotype<br>ProMed: One article reported 250 cases total of all serotypes. One article reported 10 imported cases of DENV unspecified serotype into Queensland, Australia<br>Imported cases reported on PacNet: 11 cases of DENV unspecified imported into Queensland, Australia, Australia, 4 cases of DENV unspecified from New Zealand
Jan 2016	Kiribati	Jan 2016	DENV unspecified	PacNet: 117 cases<br>Literature: Not described in the literature<br>ProMed: Not reported on ProMed<br>Imported cases: No cases were imported into another country
Jan 2016	Fiji	May 2016	DENV unspecified	PacNet: 93 cases<br>Literature: Sheel 2019 reported that 4520 suspected cases were present in Fiji in 2016<br>ProMed: One data entry reported 332 cases in May 2016; however, another reported 623 in August 2016<br>Imported cases: 3 cases were imported into New Zealand and 2 into Queensland, Australia
Feb 2016	FSM (Yap State)	May 2016	DENV unspecified	PacNet: 3<br>Literature: Not described in the literature<br>ProMed: Not reported on ProMed<br>Imported cases: No cases were imported into another country
Feb 2016	New	June 2016	DENV1	PacNet: 2

First reported (Mth/ Yr)	PICs	Latest information	Implicated virus	Summary notes and reported case numbers (confirmed/suspected/unspecified)
	Caledonia			Literature: Not described in the literature<br>ProMed: 528 cases reported<br>Imported cases: No cases were imported into another country
June 2016	Vanuatu	July 2017	DENV2	PacNet: 2950<br>Literature: Aubry 2019 reported 2 imported cases from Vanuatu into French Polynesia in Feb 2017<br>Craig 2018 reported that there was an outbreak of DEN2 during this time; however, it did not mention specific case numbers<br>ProMed: 2000 cases reported<br>Imported cases: 47 (Queensland, Australia, Aus), 4 (New Zealand), 21 (New Caledonia), 3 (French Polynesia)
Oct 2016	Republic of Palau	Dec 2017	DENV unspecified	PacNet: 440 cases<br>Literature: Not described in the literature<br>ProMed: 51 cases<br>Imported cases: No cases were imported into another country
Oct 2016	Federated States of Micronesia	Dec 2016	DENV unspecified	PacNet: 93 cases (6 laboratory confirmed)<br>Literature: Not described in the literature<br>ProMed: Not reported on ProMed<br>Imported cases: No cases were imported into another country
Nov 2016	American Samoa	April 2017	DENV2	PacNet: 885<br>Literature: Cotter 2018 3240 DENV2 cases<br>Craig 2018 reported that there was an outbreak of DEN2 during this time; however, it did not mention specific case numbers<br>ProMed: Not reported on ProMed<br>Imported cases: No cases were imported into another country
Dec 2016	Solomon Islands	June 2017	DENV1	PacNet: PacNet cases for this DENV outbreak were reported as DENV2 (12,250 DENV2 cases); however, PacNet reported imported cases of DENV1<br>Literature: No DENV1 was described in the literature at this time whilst DENV was in Solomon Islands<br>ProMed: No specific DENV1 cases for this outbreak described in ProMed<br>Imported cases reported on PacNet: 1 x DENV 1 case (Queensland, Australia, Aus), 6 (New Zealand)

First reported (Mth/ Yr)	PICs	Latest information	Implicated virus	Summary notes and reported case numbers (confirmed/suspected/unspecified)
Dec 2016	Solomon Islands	June 2017	DENV2	PacNet: 12,250 cases - on Dec 11 2016 a PacNet post stated that 60% of samples were DENV2<br>Literature: 2 literature sources mentioned this outbreak<br>Craig 2018 stated 12329 cases of DENV2, Mavian 2018 states this was DENV2; however, it does not quote any total case numbers<br>ProMed: No specific DENV2 cases for this outbreak described in ProMed<br>Imported cases: 14 x DENV2 (Queensland, Australia, Aus), 2 x DENV2 (New Zealand)
Dec 2016	Solomon Islands	June 2017	DENV3	PacNet: reported that DENV3 serotype was also identified in circulation on Dec 4 2016 as part of the larger DENV outbreak; however, the outbreak was mostly reported as a DENV2 outbreak (12,250 cases)<br>Literature: No DENV3 for this outbreak described in the literature<br>ProMed: No specific DENV3 cases for this outbreak described in ProMed<br>Imported cases: 7 x DENV3 (Queensland, Australia, Aus)
Dec 2016	Solomon Islands	June 2017	DENV unspecified	PacNet: Most PacNet cases for this DENV outbreak were reported as DENV2 (12,250 DENV2 cases) and DENV3; however, there were imported cases into other countries reported on PacNet that were unspecified<br>Literature: No DENV unspecified for this outbreak described in the literature<br>ProMed: 10,000 cases unspecified DENV cases reported in ProMed<br>Imported cases reported on PacNet: 8 x DENV unspecified (Queensland, Australia, Aus), 4 x DENV unspecified (New Zealand), 1 x DENV unspecified (Niue)
Jan 2017	New Caledonia	Sept 2017	DENV1	PacNet: reported that an outbreak of DENV1 was declared on 5 Jan 2017. The total number of cases for all DENV serotypes circulating during this time was 4467. A breakdown of each strain case number was not provided.<br>Literature:<br>Serie 2020 specific cohort of 385 cases hospitalised in New Caledonia with dengue with many also having hepatitis.<br>Craig 2018 reported that there was an outbreak of DENV1 during this time; however, did not mention specific case numbers.<br>ProMed: No specific DENV1 cases reported for this outbreak<br>Imported cases: 2 x DENV1 were imported into New Zealand
Jan 2017	New Caledonia	Sept 2017	DENV2	PacNet: DENV2 was reported to be circulating during this timeframe. The total number of cases for all DENV strains circulating during this time was 4467. A breakdown of each strain case numbers was not provided.<br>Literature: Craig 2018 reported that there was an outbreak of DEN2 during this time; however, they did not mention specific case numbers.

First reported (Mth/ Yr)	PICs	Latest information	Implicated virus	Summary notes and reported case numbers (confirmed/suspected/unspecified)
				ProMed: No specific DENV2 cases were reported for this outbreak.<br>Imported cases: 2 x DENV2 were imported into Newe Zealand.
Jan 2017	New Caledonia	Sept 2017	DENV3	PacNet: DENV3 was reported to be circulating during this timeframe. The total number of cases for all DENV strains circulating during this time was 4467. A breakdown of case numbers was not provided.<br>Literature:<br>Serie 2020– specific cohort of 385 cases of dengue hospitalised in NC with dengue + hepatitis suggests a large outbreak if hundreds of people are hospitalised with a specific complication. Serie stated that DENV3 was present in this cohort and was frequently associated with hepatitis.<br>Craig 2018 reported that there was an outbreak of DEN3 during this time; however, did not mention specific case numbers<br>ProMed: No specific DENV3 cases were reported for this outbreak<br>Imported cases: No specific DENV cases were imported from NC into another country
Jan 2017	New Caledonia	Sept 2017	DENV unspecified	PacNet: No DENV unspecified were specifically mentioned during this timeframe; however, PacNet reported imported cases<br>Literature: Serie 2020 – specific cohort of 385 cases of dengue hospitalised in NC with dengue + hepatitis suggests large outbreak if hundreds of people are hospitalised with a specific complication<br>ProMed: 1163 cases of DENV unspecified<br>Imported cases reported on PacNet: 2 x DENV unspecified were imported into New Zealand from NC
Feb 2017	Fiji	Nov 2017	DENV unspecified	PacNet: 2395 unspecified cases<br>Literature: Not described in the literature<br>ProMed: 115 in one report, 3136 in another report<br>Imported cases: 18 imported cases into Queensland, Australia and 10 into New Zealand
February 2017	Nauru	Oct 2017	DENV2	PacNet: 964<br>Literature: Craig 2018reported that there was an outbreak of DEN2 during this time; however, did not mention specific case numbers<br>ProMed: 50 cases<br>Imported cases: 8 imported into Queensland, Australia.
May 2017	Niue	July 2017	DENV4	PacNet: A post stated that in this time frame an "increase in cases" were seen in Niue, with confirmation of DENV4 serotype

First reported (Mth/ Yr)	PICs	Latest information	Implicated virus	Summary notes and reported case numbers (confirmed/suspected/unspecified)
				Literature: Not described in the literature<br>ProMed: 1 case was reported<br>Imported cases: 5 cases were imported into New Zealand
Aug 2017	Samoa	May 2018	DENV2	PacNet: 3255 cases<br>Literature: Craig 2018 reported that there was an outbreak of DEN2 during this time; however, did not mention specific case numbers<br>ProMed: 1788 cases<br>Imported cases: 103 cases imported into New Zealand, 29 imported into Queensland, Australia.
Nov 2017	Wallis and Futana	Dec 2018	DENV1	PacNet: 225 cases<br>Literature: Craig 2018 reported that there was an outbreak of DENV1 during 2017; however, they did not mention specific case numbers<br>ProMed: 229 cases<br>Imported cases: No cases were imported into another country
Jan 2018	Tonga	Oct 2018	DENV unspecified	PacNet: 69 cases<br>Literature: Not described in the literature<br>ProMed: 52 cases<br>Imported cases: 37 (New Zeland) 6 (Queensland, Australia)
Jan 2018	Fiji	June 2018	DENV unspecified	PacNet: 3437 cases, 4 deaths<br>Literature: Not described in the literature<br>ProMed: One article described 862 cases, another stated 3437.<br>Imported cases: 18 cases imported to (New Zealand), 5 cases imported into (Queensland, Australia, Aus)
Jan 2018	New Caledonia	Nov 2018	DENV1	PacNet: Total of 2003 cases of DENV during this timeframe, with 183 being DENV1<br>Mostly co-circulation of DENV 1 and DENV 2 - circulation of DENV1 - 15% of the cases<br>Literature: Not described in the literature<br>ProMed: 883 total. 740 were typed. 135 were DENV1.<br>Imported cases: No DENV1 cases imported to another country
Jan 2018	New Caledonia	Nov 2018	DENV2	PacNet: Officially declared as a DENV2 outbreak. However, co-circulation noted - mostly with DENV1.<br>Total of 2003 cases of DENV during this timeframe, with 1130 (85%) being DENV2

First reported (Mth/ Yr)	PICs	Latest information	Implicated virus	Summary notes and reported case numbers (confirmed/suspected/unspecified)
				Literature: Not described in the literature<br>ProMed: 883 total. 740 were typed. 602 were DENV2.<br>Imported cases: 3 DENV2 cases were imported into Queensland, Australia. 1 x DENV unspecified was imported into New Zealand
Jan 2018	New Caledonia	Nov 2018	DENV3	PacNet: Total of 2003 cases of DENV during this timeframe, with 2 being DENV3<br>Literature: Not described in the literature<br>ProMed: 883 total. 740 were typed. 2 were DENV3<br>Imported cases: No DENV3 cases imported to another country
Jan 2018	New Caledonia	Nov 2018	DENV4	PacNet: Total of 2003 cases of DENV during this timeframe, with 2 being DENV4<br>Literature: Not described in the literature<br>ProMed: 883 total. 740 were typed. 1 was DENV4.<br>Imported cases: No DENV4 cases imported to another country
Jan 2018	Kiribati	Oct 2018	DENV2	PacNet: 1778, 217 hospitalisations, 2 deaths<br>Literature: Not described in the literature<br>ProMed: 387 reported between Jan and May 2018<br>Imported cases: 3 imported into Queensland, Australia
Feb 2018	PNG	Dec 2018	DENV unspecified	PacNet: 7 cases reported of DENV unspecified reported in PNG<br>Literature: Not described in the literature<br>ProMed: Not reported on ProMed<br>Imported cases reported on PacNet: 19 DENV cases were imported into Queensland, Australia, Australia in this time frame, 7 cases confirmed as DENV unspecified, 4 cases were confirmed as DENV1, 1 case was confirmed as DENV2, 7 cases confirmed as DENV4
Feb 2018	PNG	Dec 2018	DENV1	PacNet: No local DENV1 cases were reported just local DENV unspecified cases, however there were DENV1 cases imported from PNG to Australia<br>Literature: Not described in the literature<br>ProMed: Not reported on ProMed<br>Imported cases reported on PacNet: 19 DENV cases were imported into Queensland, Australia, Australia in this time frame, 4 cases confirmed as DENV1

First reported (Mth/ Yr)	PICs	Latest information	Implicated virus	Summary notes and reported case numbers (confirmed/suspected/unspecified)
Feb 2018	PNG	Dec 2018	DENV2	PacNet: No local DENV2 cases were reported local DENV unspecified cases; however, there were DENV2 cases imported from Papua New Guinea to Australia<br>Literature: Not described in the literature<br>ProMed: Not reported on ProMed<br>Imported cases reported on PacNet: 19 DENV cases were imported into Queensland, Australia, Australia in this time frame, 1 case confirmed as DENV2
Feb 2018	PNG	Dec 2018	DENV4	PacNet: No local DENV4 cases were reported local DENV unspecified cases; however, there were DENV4 cases imported from Papua New Guinea to Australia<br>Literature: Not described in the literature<br>ProMed: Not reported on ProMed<br>Imported cases reported on PacNet: 19 DENV cases were imported into Queensland, Australia, Australia in this time frame, 7 cases confirmed as DENV4
Feb 2018	Vanuatu	June 2018	DENV2	PacNet: 504 cases reported, 70 confirmed<br>Literature: Craig 2018 reported that there was an outbreak of DEN2 during this time; however, they did not mention specific case numbers<br>ProMed: 246 cases reported, 44 laboratory confirmed<br>Imported cases: 6 imported into New Caledonia, 1 into New Zealand, 2 into Queensland, Australia
April 2018	American Samoa	Nov 2018	DENV2	PacNet: 1100 laboratory confirmed<br>Literature: Not described in the literature<br>ProMed: Not reported on ProMed<br>Imported cases: No cases were imported into another country
May 2018	French Polynesia	Dec 2018	DENV1	PacNet: 176<br>Literature: Not described in the literature<br>ProMed: Not reported on ProMed<br>Imported cases: 4 imported into New Zealand
June 2018	French Polynesia	June 2018	DENV2	PacNet: 2 autochthonous cases<br>Literature: Aubry 2019 described 2 autochthonous cases<br>ProMed: Not reported on ProMed<br>Imported cases: No cases were imported into another country

First reported (Mth/ Yr)	PICs	Latest information	Implicated virus	Summary notes and reported case numbers (confirmed/suspected/unspecified)
Dec 2018	Nauru	April 2019	DENV1	PacNet: 42 cases<br>Literature: Not described in the literature<br>ProMed: Not reported on ProMed<br>Imported cases: 6 imported into Queensland, Australia, Aus
Dec 2018	Republic of Palau	April 2020	DENV3	PacNet: 828 cases – first cases of DENV3 in Palau<br>Literature: Not described in the literature<br>ProMed: Not reported on ProMed<br>Imported cases: 2 imported into CNMI
Jan 2019	New Caledonia	March 2019	DENV2	PacNet: 1535 cases<br>Literature: Not described in the literature<br>ProMed: Not reported on ProMed<br>Imported cases: 1 imported into New Zealand, 1 imported in French Polynesia, 3 imported into Wallis and Futana
Jan 2019	Vanuatu	April 2019	DENV2	PacNet: 216 cases<br>Literature: Not described in the literature<br>ProMed: Not reported on ProMed<br>Imported cases: No cases were imported into another country
Jan 2019	Fiji	April 2020	DENV unspecified	PacNet: No local cases were described in PacNet; however, several imported cases were described on PacNet<br>Literature: Not described in the literature<br>ProMed: 1894 unspecified<br>Imported cases reported on PacNet:<br>DENV unspecified cases imported into New Zealand = 43<br>DENV unspecified cases imported into Queensland, Australia = 3
Jan 2019	Fiji	April 2020	DENV1	PacNet: No local DENV1 cases were described in PacNet, however several imported cases were described on PacNet<br>Literature: Not described in the literature<br>ProMed: Did not describe a DENV1 outbreak, just an unspecified outbreak of 1894 cases<br>Imported cases reported on PacNet:<br>DENV1 cases imported into New Zealand = 25

First reported (Mth/ Yr)	PICs	Latest information	Implicated virus	Summary notes and reported case numbers (confirmed/suspected/unspecified)
				DENV1 cases imported into Queensland, Australia = 19
Jan 2019	Fiji	April 2020	DENV2	PacNet: PacNet: No local DENV2 cases were described in PacNet; however, several imported cases were described on PacNet<br>Literature: Not described in the literature<br>ProMed: Did not describe a DENV2 outbreak, just an unspecified outbreak of 1894 cases<br>Imported cases reported on PacNet:<br>DENV2 cases imported into New Zealand = 4<br>DENV2 cases imported into Queensland, Australia = 7
Jan 2019	FSM (Yap State)	July 2019	DENV3	PacNet: 1661 cases (485 confirmed with NS1, 54 with IgM and 5 deaths were reported)<br>Literature: Not described in the literature<br>ProMed: Not reported on ProMed<br>Imported cases: No cases were imported into another country
Feb 2019	French Polynesia	June 2020	DENV2	PacNet: 2886 cases<br>Literature: Aubry 2019: 106 cases during a DENV2 outbreak<br>ProMed: Not reported on ProMed<br>Imported cases: 7 imported into New Zealand, 2 into Cook Islands, 1 imported into Queensland, Australia, Australia
Feb 2019	French Polynesia	February 2020	DENV3	PacNet: PacNet only reported imported cases of DENV3<br>Literature: DENV3 in French Polynesia not described in the literature at this time<br>ProMed: DENV3 in FP not reported on ProMed at this time<br>Imported cases reported on PacNet: 1 case imported into Cook islands of DENV3 from French Polynesia
Feb 2019	Cook Islands	June 2020	DENV1	PacNet: 84 cases<br>Literature: Not described in the literature<br>ProMed: Not reported on ProMed<br>Imported cases: 6 cases imported into New Zealand
Feb 2019	Cook Islands	June 2020	DENV2	PacNet: 35 cases<br>Literature: Not described in the literature<br>ProMed: Not reported on ProMed

First reported (Mth/ Yr)	PICs	Latest information	Implicated virus	Summary notes and reported case numbers (confirmed/suspected/unspecified)
				Imported cases: No imported cases into another country
Feb 2019	Cook Islands	June 2020	DENV unspec	PacNet: 233 cases<br>Literature: Not described in the literature<br>ProMed: Not reported on ProMed<br>Imported cases: 4 cases imported into New Zealand
February 2019	Wallis and Futana	June 2020	DENV2	PacNet: 77 cases<br>Literature: Not described in the literature<br>ProMed: Not reported on ProMed<br>Imported cases: No cases were imported into another country
March 2019	Tuvalu	September 2019	DENV unspecified	PacNet: 530 cases, with 215 cases RDT positives<br>Literature: Not described in the literature<br>ProMed: Not reported on ProMed<br>Imported cases: 1 imported into New Zealand, 1 imported into Kiribati
June 2019	French Polynesia	September 2019	DENV1	PacNet: 277 cases<br>Literature: Aubry 2019 reported that DENV1 transmission was present in June 2019<br>ProMed: Not reported on ProMed<br>Imported cases: 2 imported into Queensland, Australia
August 2019	Marshall Islands	June 2020	DENV3	PacNet: 3446 (1606 laboratory confirmed)<br>Literature: Not described in the literature<br>ProMed: Not reported on ProMed<br>Imported cases: No cases were imported into another country
Sept 2019	Guam	Oct 2019	DENV3	PacNet: 18 cases - These are the first confirmed locally acquired dengue cases detected in Guam in the last 75 years<br>Literature: Not described in the literature<br>ProMed: Not reported on ProMed<br>Imported cases: No cases were imported into another country
Jun 2014	American Samoa	Dec 2014	CHIKV	Pacnet: 1171 cases<br>Literature : (Wahid,2017) 2500

First reported (Mth/ Yr)	PICs	Latest information	Implicated virus	Summary notes and reported case numbers (confirmed/suspected/unspecified)
Jul 2014	Tokelau	October 2014	CHIKV	Literature : (Wahid, 2017) 200 cases<br>Pacnet : 164 cases
Jul 2014	Samoa	March 2015	CHIKV	Pacnet : 4,524 cases<br>Literature: (Wahid, 2017) 308 cases
Oct 2014	New Caledonia	Dec 2014	CHIKV	Pacnet 58 cases<br>Literature: (Nhan 2015) 29 cases
Oct 2014	French Polynesia	March 2015	CHIKV	Pacnet: 66,000 cases, 64 cases admitted to an intensive care unit, 18 deaths<br>Promed: 200000 cases
Oct 2014	Cook Islands	August 2015	CHIKV	Pacnet: 782 cases<br>Literature: (Wahid, 2017) 597 cases
Dec 2014	Kiribati	March 2015	CHIKV	Pacnet: 13,309 cases<br>Literature: (Wahid, 2017) 3000
Feb 2015	Marshall Islands	October 2015	CHIKV	Pacnet: 1,317 cases
Mar 2015	Fiji	Mar 2015	CHIKV	Literature : (Aubry 2020) 4 cases<br>Promed : 2 cases
Jun 2015	Nauru	Jun 2015	CHIKV	Pacnet: 21 cases
Oct 2015	Tuvalu	Nov 2015	CHIKV	Pacnet: 7 cases
Jan 2016	New Caledonia	Jan 2016	CHIKV	Pacnet: 2 cases
Feb 2016	Fiji	June 2016	CHIKV	Literature: (Aubry 2020) 86 cases<br>PacNet: “several cases”<br>ProMed: 41 cases
Aug 2017	Fiji	Sept 2017	CHIKV	Literature: (Aubry 2020) 2 cases
Jan 2014	New Caledonia	March 2015	ZIKV	PacNet: 7 cases<br>Literature: 2 pieces of literature<br>- Oliver :1500 cases<br>- ECDC 82 cases with 10 imported cases mentioned in the article<br>ProMed: Not reported on ProMed<br>Imported cases: No cases were imported cases were mentioned on PacNet

First reported (Mth/ Yr)	PICs	Latest information	Implicated virus	Summary notes and reported case numbers (confirmed/suspected/unspecified)
March 2015	Solomon Islands	May 2015	ZIKV	PacNet: 310<br>Literature: 5 pieces of literature<br>- Craig: 5 confirmed cases<br>- ECDC: 302 cases reported<br>- Hills: "outbreak reported" in early 2015<br>- Musso : 310 cases<br>- Mavian: stated zika was present in Solomon Island Islands in 2015<br>ProMed: In March 2016 Promed had a piece talking about the Zika Outbreak refering to a rise in GBS in 2015<br>Imported cases: 3 cases were imported from Solomon Islands to Queensland, Australia, Aus
March 2015	Vanuatu	April 2015	ZIKV	PacNet: 7<br>Literature: 5 pieces of literature<br>- Craig: 1 confirmed case in April 2015<br>- Hills: "outbreak reported"<br>- ECDC: confirmed cases in Vanuatu, unspecific numbers<br>- Musso : Unspecific number of confirmed cases reported by health officials in weeks after Cyclone Pam passed through Vanuatu in 2015<br>ProMed: in April 2015 Promed mentioned the Zika Break and the relationship with it occurring just after Cyclone Pam<br>- Mavian: states that Zika present in 2015<br>Imported cases: 1 case was imported from Vanuatu to New Caledonia
May 2015	PNG	March 2016	ZIKV	PacNet: No local cases were reported on PacNet<br>Literature: 2 pieces of literature<br>- Craig:6 confirmed cases May 2015 to March 2016<br>- Hills: described retrospective sample testing from 2015 suggests presence of Zika in 2015<br>ProMed: Not reported on ProMed<br>WHO Outbreak Report: 6 confirmed cases on retrospective samples, none of the patients positive for Zika Virus had travelled outside of PNG prior to their illness. Samples were tested from July 2014 to March 2016<br>Imported cases: Pacnet described 1 imported case from PNG to New Zealand
August 2015	Fiji	Oct 2016	ZIKV	PacNet: 15 confirmed<br>Literature: 7 pieces of literature<br>-Craig:15 confirmed cases from Aug 2015- June 2016<br>-Hills: sated that an outbreak had been reported

First reported (Mth/ Yr)	PICs	Latest information	Implicated virus	Summary notes and reported case numbers (confirmed/suspected/unspecified)
				- Kama: 13 confirmed cases July 2015- March 2016<br>- Sheel: 583 zika like illnesses after the cyclone 2016<br>-Paixo: Reported Zika cases in 2016<br>-Musso reported cases however exact numbers were not availaboratoryle in 2015<br>-Mavian: reported that Zika was present in 2015<br>ProMed: 17 cases from March - June 2016<br>Imported cases: 10 imported into New Zealand, 8 imported into Queensland, Australia, Aus
September 2015	Samoa	May 2016	ZIKV	PacNet: 167 suspected cases, 24 confirmed cases<br>Literature: 6 pieces of literature<br>- Craig: 24 confirmed cases Sept 2015- May 2016<br>- Hills: stated than an outbreak had been reported<br>- Samrasekera: reported active transmission as of Feb 2016<br>- Paixo:reported an undetermined number of cases of Zika in Samoa in 2016<br>- Musso: reported cases in Samoa in 2015<br>- Mavian: statedthat zika was present in 2015 and 2016<br>ProMed: 11 cases active in March 2016<br>Imported cases: 22 cases imported into New Zealand, 1 case imported into Queensland, Australia
Dec 2015	Cook Islands	Dec 2015	ZIKV	PacNet: Reported that Zika cases had been reported for the first time in Cook Island, no numbers were stated<br>Literature: Not described in the literature<br>ProMed: Not reported on ProMed<br>Imported cases: No cases were imported into another country
Jan 2016	Tonga	June 2016	ZIKV	PacNet: 2420 cases<br>Literature: 3 pieces of literature<br>- Craig: 2 confirmed cases Jan 2016- April 2016<br>- Hills: reported cases however no dtaes<br>- Mavian: reported that Zika was present<br>ProMed: Jan 2016 reported that 2000 suspected cases, with Australia confirming a case in a traveller returning to Queensland, Australia from Tonga<br>Imported cases: No cases were imported into another country
Jan 2016	American Samoa	Feb 2017	ZIKV	PacNet: 1004 suspected, 58 confirmed, 33 pregnant confirmed cases<br>Literature: 4 pieces of literature

First reported (Mth/ Yr)	PICs	Latest information	Implicated virus	Summary notes and reported case numbers (confirmed/suspected/unspecified)
				- Craig 23 confirmed cases from Jan 2016 to April 2016<br>- Healy: 756 suspected, 51 laboratory confirmed, between Jan 2015- July 2015<br>- Samarasekera reported that there was active transmission in Feb 2016<br>- Mavian stated that Zika was present in 2016<br>ProMed: 2 Promed reports – one stating that from Feb 2016 – November 2016 estimated 1000 cases, another report stating that there had been 25 confirmed cases<br>Imported cases: No cases were imported into another country
Feb 2016	Republic of Marshall Islands	April 2016	ZIKV	PacNet: 34 suspected cases, 2 confirmed<br>Literature: 3 pieces of literature<br>- Craig: 2 confirmed cases Feb 2016- April 2016<br>- Hills: reported that there were cases present<br>- Mavian stated that zika was present in 2016<br>ProMed: 2 cases reported<br>Imported cases: 1 case was imported into Australia
Feb 2016	FSM	Dec 2016	ZIKV	PacNet: 238 cases, 27 confirmed, 20 pregnant confirmed cases<br>Literature: 2 pieces of literature<br>- Craig- 23 confirmed cases in Feb 2016, and added that the outbreak was still ongoing in Nov 2016<br>- Hills: reported cases however no numbers or dates<br>ProMed: Reported that there was local transmission however no numbers stated<br>Imported cases: No cases were imported into another country
March 2016	New Caledonia	March 2016	ZIKV	PacNet: 1 local case<br>Literature: Craig : 82 confirmed cases<br>ProMed: Not reported on ProMed<br>Imported cases: No cases were imported into another country
Nov 2016	Palau	Nov 2016	ZIKV	PacNet: 1 laboratory confirmed case<br>Literature: 3 pieces of literature<br>- Craig: 1 confirmed case in Nov 2016<br>- Hills: cases were reported, nil dates or exact figures<br>- Mavian: stated that Zika was present in 2016<br>ProMed: Reported that there was 1 locally acquired case in Nov 2016<br>Imported cases: No cases were imported into another country

First reported (Mth/ Yr)	PICs	Latest information	Implicated virus	Summary notes and reported case numbers (confirmed/suspected/unspecified)
2016 (no specific months listed)	Solomon Islands	2016	ZIKV	PacNet: No cases reported<br>Literature: Paixo reported that there were Zika cases in Solomon Islands in 2016, no specific dated or numbers were reported<br>ProMed: Not reported on ProMed<br>Imported cases: 1 case was imported from Solomon Islands to Queensland, Australia, Aus
2016 (no specific months listed)	French Polynesia	2016	ZIKV	PacNet: No reported cases<br>Literature: Musso reported 42 asymptomatic blood donor patients found to have Zika through a study looking at blood transfusions, no specific dates given<br>ProMed: Not reported on ProMed<br>Imported cases: No cases were imported into another country
2016 (no specific months listed)	Kiribati	2016	ZIKV	PacNet: No reported cases<br>Literature: Mavian stated that Zika was present in Kiribati in 2016, no specific dates or numbers mentioned<br>ProMed: Not reported on ProMed<br>Imported cases: No cases were imported into another country
Jan 2017	Republic of Marshall Islands	Jan 2017	ZIKV	PacNet: Reported that a State of Health Emergency was declared for Republic of Marshall Islands in Jan 2017 for Zika<br>Literature: Not described in the literature<br>ProMed: Not reported on ProMed<br>Imported cases: No cases were imported into another country
June 2018	American Samoa	Nov 2018	ZIKV	PacNet: 26<br>Literature: Not described in the literature<br>ProMed: Not reported on ProMed<br>Imported cases: No cases were imported into another country
